# Neoadjuvant chemoradiation and surgical excision versus definitive radiotherapy for locally advanced cervix uteri carcinoma: in terms of early and late complications and locoregional recurrence

**DOI:** 10.1186/s43046-025-00292-0

**Published:** 2025-06-07

**Authors:** Hisham Khalifa, Mohamed Ayaty, Reham Oreaba, Reem Emad, Mohamed Salama, khaled Elsebahy, Wael A. Wahab Ghoniem

**Affiliations:** National cancer institute Egypt, cairo, Egypt

**Keywords:** Definitive radiotherapy, Cervix, Neoadjuvant chemoradiation, Adjuvant hysterectomy

## Abstract

**Objectives:**

Following external beam radiation therapy (EBRT) with concurrent chemotherapy, we analyzed the benefits of surgical resection for locally advanced cervical carcinoma in terms of the frequency and severity of complications and disease-free survival, including cases of adjuvant hysterectomy after failure of resolution post-brachytherapy.

**Patient and methods:**

Retrospective analysis was utilized to determine the eligibility of 145 cases treated at the National Cancer Institute between January 2015 and June 2021. Of those, 17 patients did not match the requirements, and 8 patients declined to take part in the study. Depending on the major treatment technique, 120 FIGO stage IB3–FIGO stage IVA cervical cancer patients were split into two equal groups of 60 patients each. Sixty patients (50%) received neo-adjuvant EBRT and concurrent platinum-based chemotherapy followed by hysterectomy (group A) and 60 (50%) received definitive radiotherapy only (group B).

**Results:**

The age at diagnosis of patients was similar, with a mean of 52.5 (range 34–77) and 53.4 (range 25 81) years in group A and group B, respectively (*P* = 0.675). Majority of the cases in both groups were pathologically squamous cell carcinomas (88.3% in group A and 83.3% in group B) and of grade II differentiation (73.7% in group A and 71.2% in group B). Majority of cases in both groups being FIGO stage II (45% in group A and 40% in group B) and FIGO stage III (40% in group A and 43.3% in group B). Only 17 patients (28.3%) in group A had postoperative complications, while 37 patients (61.7%) in group B suffered from post-treatment complications (*P* value < 0.001). In group B, 14 patients (23.3%) failed to show complete remission of the disease after completion of treatment, with a mean residual disease of 4.3 cm in diameter (range 2–6 cm), either local or nodal. Salvage hysterectomy post-definitive radiotherapy was done for 8 patients with residual disease (13.3%). In group A, 48 patients had no recurrence during follow-up (80%), while 11 of the patients had either locoregional or metastatic recurrences, or both (18.3%). DFS was comparable between both groups (*P* = 0.493), excluding 23.3% of group B where failure of complete remission of the disease after completion of treatment barred the patients from the disease-free calculations. The 1-year DFS was 88.1% in group A and 82.6% in group B, while the 3-year DFS was 74.1% in group A and 70.1% in group B.

**Conclusion:**

There was no difference in disease-free survival or the incidence of locoregional and metastatic recurrence between patients with cervical cancer who had surgery and those who received brachytherapy following EBRT and concomitant chemotherapy. In almost 50% of cases, the surgical patients showed full pathological recovery.

## Introduction

Cervical cancer remains a significant global health challenge, particularly in cases where the disease is diagnosed at a locally advanced stage. Standard treatment for locally advanced cervical cancer (LACC) typically involves definitive chemoradiation rather than upfront surgery. However, in specific cases where surgery is still considered an option, neoadjuvant therapy can improve outcomes by reducing tumor burden, controlling microscopic disease, and increasing the likelihood of complete resection.

There is ongoing debate regarding the optimal neoadjuvant approach: neoadjuvant radiotherapy versus neoadjuvant chemotherapy. While both strategies have been explored, neoadjuvant radiotherapy is often preferred due to its enhanced local control, superior tumor shrinkage, and better preservation of surgical feasibility. Radiotherapy effectively reduces tumor volume and treats regional lymph nodes simultaneously, whereas chemotherapy primarily targets systemic disease with less predictable local response rates. Moreover, radiation can directly induce tumor necrosis and improve resectability, whereas chemotherapy’s impact on tumor regression is variable, potentially leaving residual viable tumor cells [1].

The current evidence points to a golden standard in treatment of locally advanced cervical cancer which consists of EBRT with concomitant platinum-based chemotherapy followed by brachytherapy, a treatment protocol referred to as ‘definitive radiotherapy’ [1].

Weijia Lu et. al. (2021) conducted a recent meta-analysis on the benefits of adjuvant hysterectomy, whether or not prior brachytherapy was received after EBRT, revealing an overall analysis indicating improved overall survival and disease-free survival with the use of adjuvant hysterectomy after concurrent chemoradiotherapy, however failing at the level of subgroup analysis based on similar treatment protocols to demonstrate any significant benefit of hysterectomy in locally advanced cervical cancer. Yet, the results indicated that the recurrence rate may be higher in patients receiving chemoradiotherapy without hysterectomy [2].

Furthermore, findings from a large Chinese cervical cancer database indicated that preoperative radiotherapy significantly reduces deep cervical stromal invasion and vascular space invasion, which are critical factors influencing disease progression and prognosis (Zhang et al., 2019). However, the study also noted that preoperative radiotherapy does not significantly reduce lymph node positivity, parametrial involvement, or positive surgical margins, suggesting that while it enhances certain pathological outcomes, its effect on complete disease clearance may be limited [3].

In support of radical surgery post-neoadjuvant therapies, the removal of residual tumor, pathological assessment for the treatment response and the prognostic value is advocated. Residual tumor after neoadjuvant chemoradiotherapy represents an independent prognostic factor, showing a better prognosis for patients with complete with respect to partial pathological response. Contrariwise, the complications rate of applying the three-modality approach remains an issue, as well as the reduction of available treatments for the recurrent disease [4].

## Materials and methods

Retrospective analysis was utilized to determine the eligibility of 145 cases treated at the National Cancer Institute between January 2015 and June 2021. Of those, 17 patients did not match the requirements, and 8 patients declined to take part in the study.

**Fig. 1:**
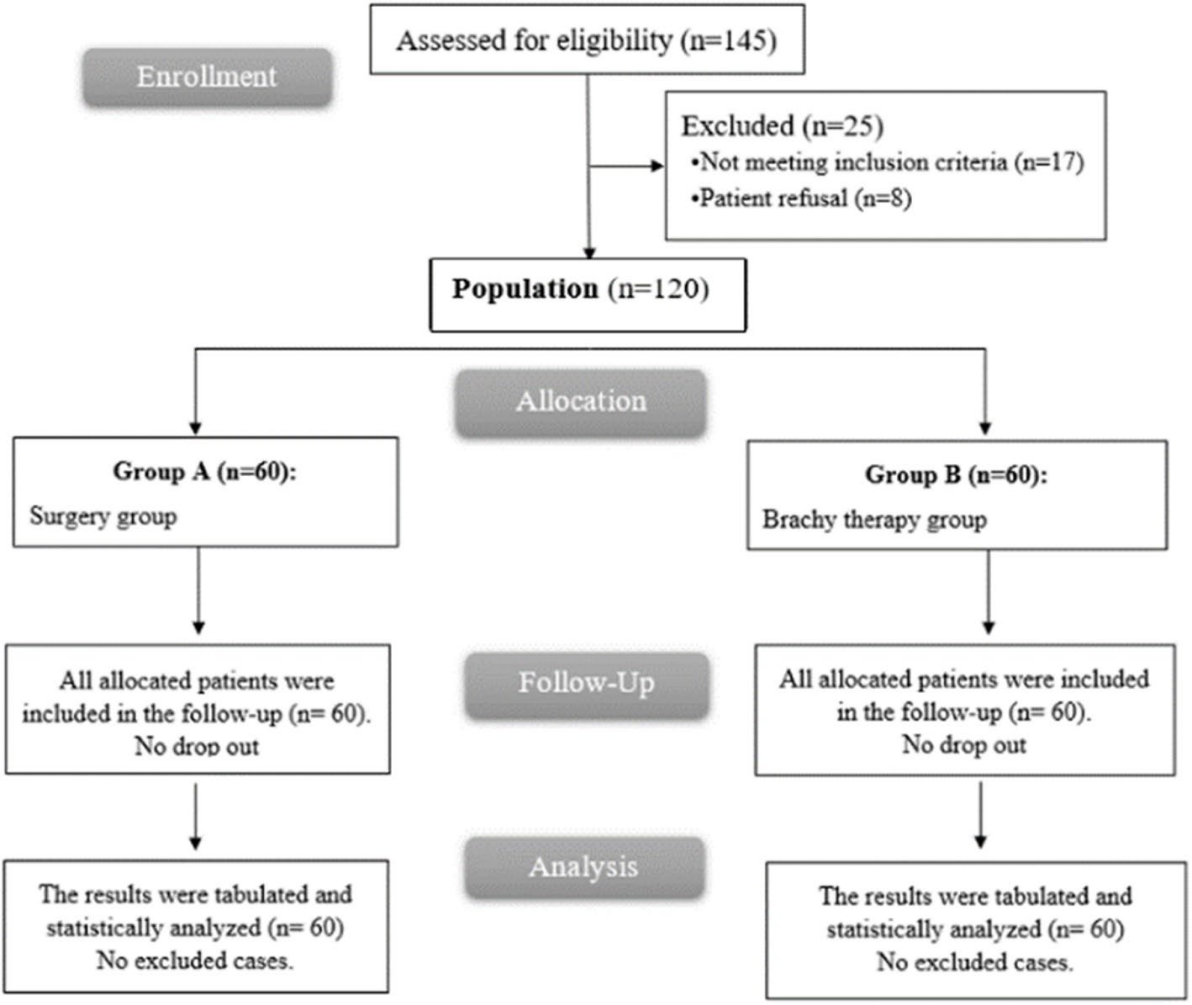
Population analysis

120 FIGO stage IB3–FIGO stage IVA cervical cancer patients were included in this retrospective research. These patients were acquired at the National Cancer Institute's Gynecological Oncology and Radiation Oncology departments in Cairo, Egypt, depending on the major treatment technique, the remaining patients were split into two equal groups of 60 patients each. Every patient that was assigned was tracked down and statistically examined (Fig. 1).

The study population was divided into 2 groups according to the type of treatment: 60 patients (50%) received neo-adjuvant EBRT and concurrent platinum-based chemotherapy followed by radical surgery (group A) and 60 (50%) received definitive radiotherapy only (group B).

Patients were allocated to different strategies according to internal protocols including anatomic and pathologic possibility and availability of brachytherapy.

Inclusion criteria were patients with positive biopsy for cervical carcinoma, FIGO stage IB3–IVA and received standard EBRT with concurrent platinum-based chemotherapy. Patients who underwent radical surgery following definitive radiotherapy were included as part of the group B analysis, as management of residual or recurrence.

Pre-treatment work-up included clinical examination under anesthesia, chest and abdominal radiography and pelvic MRI for locoregional and metastatic workups. Preoperative pelvic MRI was performed for group A patients, with comparative analysis to pathological outcomes of resection done. Cystoscopy and proctoscopy were performed in selected cases. Preoperative ureteric stenting was done in selected cases, and comparative analysis of outcome regarding complications done.

Platinum-based chemotherapy was 40 mg/m^2^ of cisplatin per week or 20 mg/m^2^ (2-h intravenous infusion, on days 1–4 and 26–30 of treatment) in both groups over the course of the EBRT.

In Groub A, neo-adjuvant treatment was concomitant platinum-based chemotherapy plus a median dose of 50 Gy of external beam radiation. At 4 to 6 weeks after the end of chemoradiotherapy, patients were evaluated for clinical response according to RECIST criteria with clinical examination and MRI and submitted to radical hysterectomy defined according to the Querleue Morrow classification. Surgery was performed by an open approach. Extension of radical hysterectomy was pre-operatively planned according to clinical response, and tailored during the procedure taking into account intraoperative findings.

In group B, definitive radiotherapy consisted of concurrent platinum-based chemotherapy plus a median of 50 Gy of external beam radiation divided to a median of 25 fractions with median 7 Gy of vaginal brachytherapy. In 5 cases a median of 20 Gy 3D boost IMRT was delivered in place of brachytherapy.

Frequency and severity of complications were compared between both groups. In group A, treatment complications were also classified according to Clavien-Dindo classification, according to severity and management required.

Follow-up was done with clinical examination, examination under anesthesia, pelvic MRI, and chest and abdomen radiography, to detect locoregional or metastatic recurrence. A minimum of 1 year follow-up period was required, starting from the date of surgery in group A and from the date of image confirmed resolution of disease in group B.

### Statistical analysis

Statistical analysis was done by SPSS v26 (IBM Inc., Chicago, IL, USA). Quantitative variables were presented as mean and standard deviation (SD) and compared between the two groups utilizing unpaired Student’s *t* test. Qualitative variables were presented as frequency and percentage (%) and were analyzed utilizing the Chi-square test or Fisher’s exact test when appropriate. A two tailed *P* value < 0.05 was considered statistically significant. Disease-free survival (DFS) was calculated from the end of treatment to the date of relapse or until the date of the last follow-up. Medians and life tables were computed using the product limit estimate by Kaplan–Meier methods.

### Ethical approval

The study was approved by Cairo University's National Cancer Institute Ethics Committee. At the time of their admission, every participant consented in writing to the use of their data for medical study. The study was conducted in accordance with the Helsinki Declaration.

## Results

The clinic-pathological characteristics of the patients admitted to both groups are listed in Table 1.

**Table 1:** Clinical-pathological comparison of patients admitted to both groups

		**Group A** ***n*** **= 60**	**Group B** ***n*** **= 60**	***P*** ** value**
**Age (years)**	Mean ± SD	52.5 ± 10.4	53.4 ± 12.0	0.675
	Range	34–77	25–81	
**Age g**	≤ 52 years	33 (55.0)	27 (45.0)	0.273
	> 52 years	27 (45.0)	33 (55.0)	
**Comorbidity**	No	30 (50.0)	36 (60.0)	0.271
	Yes	30 (50.0)	24 (40.0)	
**Hypertension**	No	36 (60.0)	44 (73.3)	0.121
	Yes	24 (40.0)	16 (26.7)	
**Diabetes Mellitus**	No	46 (76.7)	47 (78.3)	0.827
	Yes	14 (23.3)	13 (21.7)	
**Cardiac**	No	60 (100.0)	57 (95.0)	0.244
	Yes	0 (.0)	3 (5.0)	
**Others**	No	56 (93.3)	53 (88.3)	0.343
	Yes	4 (6.7)	7 (11.7)	
**Pathology**	SCC	53 (88.3)	50 (83.3)	0.222
	Adenocarcinoma	4 (6.7)	9 (15.0)	
	Undifferentiated Carcinoma	3 (5.0)	1 (1.7)	
**Grade**	I	0 (.0)	1 (1.7)	0.607
	II	42 (73.7)	42 (71.2)	
	III	15 (26.3)	16 (27.1)	
**Stage**	IB3	7 (11.7)	4 (6.7)	0.380
	II	27 (45.0)	24 (40.0)	
	III	24 (40.0)	26 (43.3)	
	IVA	2 (3.3)	6 (10.0)	
**Max dim**	≤ 5	33 (55.0)	27 (45.0)	0.273
	> 5	27 (45.0)	33 (55.0)	
**Maximum diameter(cm)**	Mean ± SD	5.2 ± 1.4	5.4 ± 1.4	0.401
	Range	2–10	2–8	
**Extension**				
** Upper vagina**	No	35 (58.3)	37 (61.7)	0.709
	Yes	25 (41.7)	23 (38.3)	
** Unilateral parametrium**	No	37 (61.7)	43 (71.7)	0.245
	Yes	23 (38.3)	17 (28.3)	
** Bilateral parametrium**	No	39 (65.0)	48 (80.0)	0.066
	Yes	21 (35.0)	12 (20.0)	
** Lower vagina**	No	60 (100.0)	56 (93.3)	0.119
	Yes	0 (.0)	4 (6.7)	

The age at diagnosis of patients was similar, with a mean of 52.5 (range 34–77) and 53.4 (range 25–81) years in group A and group B, respectively (*P* = 0.675).

While 50% of patients in group A had comorbidities before treatment, 40% of patients in group B had comorbidities including hypertension, diabetes mellitus, ischemic heart disease, hypothyroidism and anxiety disorders (*P* = 0.271).

Majority of the cases in both groups were pathologically squamous cell carcinomas (88.3% in group A and 83.3% in group B) and of grade II differentiation (73.7% in group A and 71.2% in group B). Both type and differentiation of tumors were comparable in both groups (*P* = 0.222 and *P* = 0.607, respectively).

The FIGO stage of disease at diagnosis was comparable as well between both groups (*P* = 0.38), with majority of cases in both groups being FIGO stage II (45% in group A and 40% in group B) and FIGO stage III (40% in group A and 43.3% in group B).

The size and the extension of the primary cervical tumor were studied in both groups to further delineate the effectiveness of each treatment modality towards particular cases, and were initially comparable between both groups (Table 1).

### Complications of treatment

Table 2 shows post-treatment complications in both groups. Only 17 patients (28.3%) in group A had postoperative complications, while 37 patients (61.7%) in group B suffered from post-treatment complications (*P* value < 0.001).

**Table 2:** Complications of treatment

	**Group A** ***n* = 60**	**Group B** ***n* = 60**	***P*** **value**
**Urinary complications, *n*** **(%)**			
**Urinary tract infections**	2 (3.3)	0	0.495
**Recurrent cystitis**	2 (3.3)	19 (31.6)	< 0.001
**Ureteric backpressure**	3 (5)	4 (6.6)	1.00
**Urinary tract injuries/fistula**	2 (3.3)	1 (1.67)	1.00
**Hematuria**	0	1 (1.67)	1.00
**Total**	9 (15)	25 (41.7)	0.001
**Gastrointestinal, *n*** **(%)**			
**Chronic constipation**	0	2 (3.3)	0.495
**Chronic diarrhea**	0	6 (10)	0.027
**Proctitis**	0	4 (6.6)	0.118
**Rectovaginal fistula**	0	4 (6.6)	0.118
**Total**	0	16 (26.7)	< 0.001
**Lympho-vascular, *n*** **L(%)**			
**Severe bleeding**	1 (1.67)	1 (1.67)	1.504
**Lower limb edema**	0	2 (3.3)	0.495
**Deep venous thrombosis**	2 (3.3)	0	0.495
**Total**	3 (5)	3 (5)	1.320
**Chronic pain, *n*** **(%)**			
**Lower abdominal**	0	12 (20)	< 0.001
**Back**	0	7 (11.67)	0.013
**Other**	0	2 (3.3)	0.495
**Total**	0	21 (35)	< 0.001
**Vaginal, *n*** **(%)**			
**Stenosis**	0	15 (25)	< 0.001
**Miscellaneous, *n*** **(%)**			
**Wound infections**	3 (5)	0	0.243
**Collection/abscess**	2 (3.3)	2 (3.3)	1.381
**Incisional hernia**	1 (1.67)	0	1.00
**S1 fracture (with no related soft tissue mass)**	0	1 (1.67)	1.00
**Total**	6 (10)	3 (5)	0.490
**Total *n*** **(%)**	17 (28.3)	37 (61.7)	< 0.001

Urinary tract complications in group A included urinary tract infections (3.3%), cystitis (3.3%), ureteric back pressure (5%), ureteric injuries (1.67%), and urinary bladder injuries (1.67%).

Preoperative ureteric stenting was done in 25 patients (35%), all of whom had no postoperative urinary tract complications.

While in group B, urinary tract complications included radiation cystitis (31.6%), ureteric backpressure (6.6%), ureterovaginal fistula (1.67%), and macrohematuria (1.67%).

Gastrointestinal, vaginal complications, and chronic pain were predominantly in group B patients, while lympho-vascular and miscellaneous complications were equivocal in both groups.

Postoperative complications in group A patients were classified according to the Clavien-Dindo classification of postoperative complications (Table 3).

**Table 3:** Clavien-Dindo classification of group A postoperative complications

**Grade**	**Complications**	**Frequency** ***n*** **= 60 (%)**	**Treatment**
**II**	Wound infection	3 (5)	Wound culture, oral antibiotics
	Urinary tract infections	4 (6.6)	Urine culture, oral antibiotics
	Deep venous thrombosis	2 (3.3)	Therapeutic anticoagulation
	Blood loss	1 (1.67)	Blood transfusion
**IIIa**	Ureteric backpressure	3 (5)	Ureteric stenting
	Collections	2 (3.3)	Ultrasound guided drainage
**IIIb**	Incisional hernia	1 (1.67)	Follow-up
**IV**	Postoperative hypertension	1 (1.67)	ICU admission
**V**	Intraoperative ureteric injury, intra-abdominal urinary leakage, Sepsis, death	1 (1.67)	ICU admission

In group A, 9 patients (15%) had high risk pathological criteria including positive lymph node metastasis, close or positive margins and lympho-vascular infiltration. 4 patients (6.6%) had positive lymph node metastasis, 2 patients (3%) had positive lympho-vascular infiltration without nodal metastasis and 3 patients (5%) had close or positive margins. Six patients (10%) received adjuvant chemotherapy and 5 patients remained under close follow-up.

In group B, 14 patients (23.3%) failed to show complete remission of the disease after completion of treatment, with a mean residual disease of 4.3 cm in diameter (range 2–6 cm), either local or nodal (Table 4).

**Table 4:** Site and complications of residual disease in group B

	**Frequency** ***n*** **= 14**
Site of residual	
Cervix	4
Cervix and parametrium	6
Extending to UB	2
Pelvic lymph nodes	1
Para aortic lymph nodes	1
Complications of residual	
Bleeding	4
Ureteric backpressure	2
Vertebral invasion	1

One patient (1.67%) had rapidly progressive disease leading to death before management of residual. Two patients (3.3%) could not receive further treatment post-definitive radiotherapy due to poor performance status and were put on best supportive care. One patient (1.67%) had extensive bleeding due to residual disease, received 500 cGy/5 sessions hemostatic radiotherapy followed by chemotherapy. Two patients (3.3%) received chemotherapy post-definitive radiotherapy with disease-free follow-up.

Salvage hysterectomy post-definitive radiotherapy was done for 8 patients with residual disease (13.3%). Four patients were found to have complete pathological response, with disease-free follow-up. The other 4 patients however were found to have positive pathological residual, one of which developed a vaginal stump recurrence after 14 months, later excised and found to be pathologically positive for recurrence, while the other 3 had disease-free follow-up.

Of the remaining 46 patients in group B who had complete radiological resolution post-definitive radiotherapy, 33 patients had disease-free follow-up (55%), 8 patients developed locoregional recurrence (13.3%) after an average of 10.2 months (range 5–21 months), 2 of which also developed metastatic recurrence. While 5 patients developed metastatic recurrence (8.3%), after an average of 9 months (range 6–16 months).

Salvage hysterectomy was done for 2 patients with local recurrence (Table 5).

**Table 5:** Site and treatment of recurrent disease in group B

	**Frequency** ***n*** ** = 13**
Site of recurrence	
Cervix	3
Lateral pelvic wall	3
Pelvic/paraaortic lymph nodes	2
Pulmonary	6
Bone	2
Treatment of recurrence	
Chemotherapy	9
Salvage hysterectomy	2
Supportive care	1
Follow-up	1

In group A, 48 patients had no recurrence during follow-up (80%), while 11 of the patients had either locoregional or metastatic recurrences, or both (18.3%). Locoregional recurrence developed in 7 patients after an average of 9.7 months (range 3–15 months), 4 later developed metastatic recurrence, after an average of 10.75 months (range 8–15 months). While 4 other patients developed metastatic recurrence only after an average of 14.75 months (range 6–27 months) (Table 6).

**Table 6:** Characteristics of recurrent disease in group A

	**Frequency** ***n*** ** = 11**
Site of recurrence	
Operative bed/vaginal stump	7
Reaching the lateral pelvic wall	2
Pelvic/paraortic lymph nodes	2
Pulmonary	4
Bone	1
Cervical/mediastinal lymph nodes	1
Peritoneal	1
Complications of recurrence	
Chronic pain	6
Ureteric backpressure	5
Treatment of recurrence	
Chemotherapy (curative/palliative)	9
Best supportive care	2
Nephrostomy	5

### Disease-free survival (DFS)

DFS was comparable between both groups (*P* = 0.493), excluding 23.3% of group B where failure of complete remission of the disease after completion of treatment barred the patients from the disease-free calculations.

**Fig. 2:**
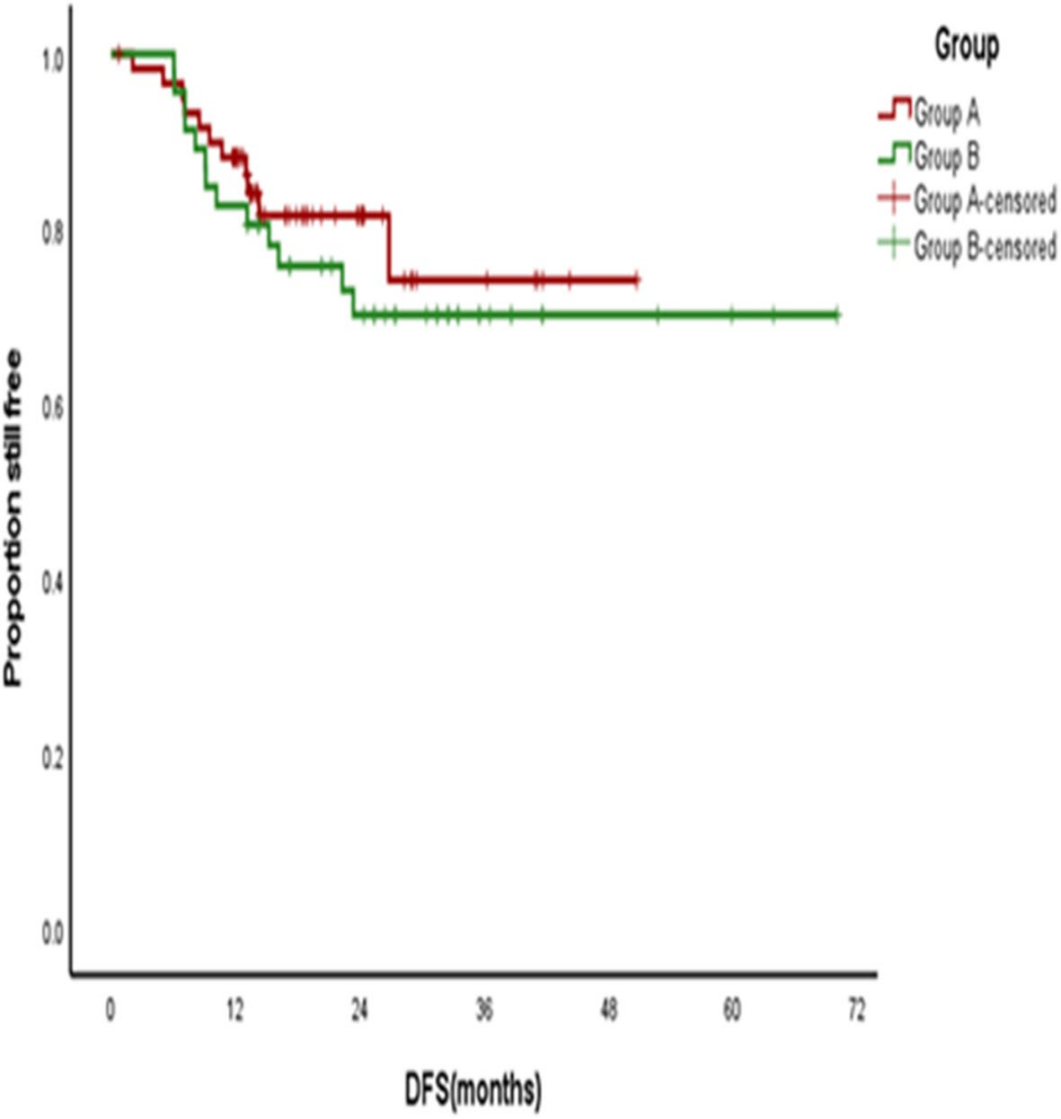
DFS in both groups

The 1-year DFS was 88.1% in group A and 82.6% in group B, while the 3-year DFS was 74.1% in group A and 70.1% in group B (Table 7 and Fig. 2).

**Table 7:** Disease-free survival in both groups

**DFS %**						
	***n***	**6 months**	**1 year**	**2 years**	**3 years**	***P*** ** value**
Group A	60	94.9	88.1	81.5	74.1	0.493
Group B	46	95.7	82.6	70.1	70.1

## Discussion

In the treatment of locally advanced cervical cancer, the three-modality approach of neoadjuvant chemoradiotherapy followed by radical hysterectomy was proposed in 1970 and subsequently reconsidered. in prospective randomized studies. However, the main concern remains the combined morbidity of the three modalities and definitive radiotherapy, which therefore remains the focus of treatment [5].

In the last 20 years, this multimodality approach was reevaluated and the disease-free and overall survival rates were at least analogous to those of definitive radiotherapy, with a different morbidity spectrum [6, 7]. In particular, chronic toxic effects (e.g., proctitis, cystitis, hydronephrosis) were less in patients undergoing surgery possibly because of substitution of brachytherapy with completion surgery [8].

The aim of this study is to compare the outcomes of the patients who underwent surgery with those who received brachytherapy, after EBRT with concurrent chemotherapy, in terms of early and late complications and locoregional recurrence.

This retrospective analysis included 120 FIGO stage IB3–FIGO stage IVA cervical cancer patients accrued between January 2015 and June 2021 at the department of Gynaecological Oncology and department of Radiation Oncology of the National Cancer Institute, Cairo, Egypt.

The study population was divided into 2 groups according to the type of treatment: 60 patients (50%) received neo-adjuvant EBRT and concurrent platin-based chemotherapy followed by radical surgery (group A) and 60 (50%) received definitive radiotherapy (group B). Fanfani et al. (2009) have reported that this approach could be administered in patients with stage IIIB cervical cancer with an acceptable rate of complications and with a survival outcome similar to that reported for standard CT-RT, allowing the assessment of pathological response with its implication on clinical outcomes [8].

According to our research, 37 patients (61.7%) in group B experienced post-treatment complications, whereas 17 patients (28.3%) in group A, the surgery group, experienced post-operative complications. *P* value < 0.001 indicates a considerably greater rate of post-treatment problems overall in group B, the group receiving radical radiation. Landoni et al. (2017) stated that complications were statistically significantly greater in the surgical group, which is contrary to our findings [9].

Urinary, gastrointestinal, chronic pain, and vaginal problems were significantly more common in group B compared to group A in our research. Urinary tract infections, ureteric backpressure, urinary tract injuries or fistulas, and haematuria did not substantially vary between the two groups, while group B experienced a greater incidence of recurrent cystitis. In terms of gastrointestinal problems, group B reported more cases of chronic diarrhoea than group A, but there was no significant difference in proctitis, rectovaginal fistula, or chronic constipation between the two groups.

A matched retrospective comparison of the treatment outcomes between patients treated with similar chemoradiation followed by brachytherapy and those treated with EBRT plus radical hysterectomy was carried out by Cetina et al. (2009) [10]. Urinary problems were more common in the surgery group, which is different from our findings. On the other hand, chronic proctitis was more evident in the brachytherapy group which is similar to our findings.

According to our research, urinary tract complications in group A included urinary tract infections (3.3%), cystitis (3.3%), ureteric back pressure (5%), ureteric injuries (1.67%), and urinary bladder injuries (1.67%). Preoperative ureteric stenting was done in 25 patients (35%), all of whom had no postoperative urinary tract complications. But, according to Cetina et al. (2013), among the acute complications in the surgical arm, 1.5% had wound dehiscence, another 1.5% had infection in the surgical site, 3.4% had vascular laceration, 1.5% had a urethral rupture, and 2.3% had a section of the ureter. Six individuals had infection 30 days following surgery due to late toxicity in the operative arm; 3.4% of these patients developed unilateral lymphocysts that needed to be treated with percutaneous draining. Furthermore, 2.3% of patients underwent surgery and double J-stent placement to address uretero-cutaneous fistulae [11].

In a related research, Wang et al. (2014) compared 119 patients in the radical radiation group and 121 in the surgery post-EBRT group. The results indicated that there was no overall statistically significant difference in the late complications rate between the two groups. On the other hand, radiation enteritis was more common in the definitive radiotherapy group and leg edema was statistically substantially greater in the surgical group [12].

Similar to our findings, Fanfani et al. (2016) showed statistically significant higher rates of early gastrointestinal and urinary problems in the radiation group as compared to the surgical group. There was no discernible difference in late problems between the two groups. Vascular problems, however, were limited to the surgical group [13].

In a systematic review done by Kol K.V, et al. a total of 202 complications related to hysterectomy were registered in 578 patients treated with chemoradiation and surgery (35%), compared to the lower complications rate in the surgical group in this study, where 17 patients (28.3%) only were registered out of 60 patients and this could be attributed to the lower number of patients in our study [14].

According to our research, there was no discernible difference in the disease-free survival between the group A and group B groups. Group A and group B had 1-year DFS of 88.1% and 82.6%, respectively, and 3-year DFS of 74.1% and 70.1%, respectively.

Similar to our results, Cetina et al. (2009) showed that progression-free and overall survival are similar with a predicted 5-year survival of 78% at a maximum follow-up of 60 months, median follow-up of 26 (2–31) and 22 (3–27) months for the surgical and conventional treatment groups. Eight patients in each group experienced a relapse and eventually passed away [10].

Similar to our results, Cetina et al. (2013) found that progression-free survival and overall survival were comparable in both arms after a median follow-up period of 36 months (3–80): DFS rates in the surgical arm were 71.7%, while those in the brachytherapy arm were 74.8% [11].

Additionally, Fanfani et al. (2016) found no statistically significant difference in the DFS between patients who had radical hysterectomy following neoadjuvant chemoradiation and those who had definitive radiotherapy [12].

In line with our results, Landoni et al. (2017) conducted research comparing brachytherapy with radical surgery for the treatment of stage IB–IIA cervical cancer. They discovered no statistically significant difference in the overall survival rates between the two procedures [9].

In addition, there was no statistically significant difference in progression-free survival between patients receiving brachytherapy and those undergoing hysterectomy, whether simple or radical, according to Hass et al.’s retrospective analysis of 121 patients with locally advanced cervical cancer following EBRT [13].

In order to eliminate treatment protocol differences because brachytherapy affects patients’ total radiation dose, Lu et al. (2021) conducted a meta-analysis of 14 studies comparing the role of hysterectomy post-chemoradiation for locally advanced cervical cancer versus chemoradiation alone. For 4 of these studies, a subgroup analysis was provided. There was no statistically significant difference in the disease-free survival between the two groups, according to the brachytherapy-based subgroup analysis [2]. However, Wang et al. (2014) on contrary our research results, found that the surgical group had a higher 3-year overall survival (94.9% against 84.6%, *P* = 0.011) and 3-year progression-free survival (91.0% versus 81.8%, *P* = 0.049) than the radical radiation group [15].

## Conclusion

Locally advanced cervical cancer patients who underwent surgery following EBRT with concurrent chemotherapy have less complications compared to those who received definitive radiotherapy. *P* value < 0.001 indicates a considerably greater rate of post-treatment problems overall in group receiving definitive radiotherapy. Moreover, there was no difference in disease-free survival and locoregional and metastatic recurrence between both groups.

## Data Availability

No datasets were generated or analysed during the current study.

## References

[1] Serarslan A, Meydan D, Yildiz RE. Radical radiotherapy of locally advanced cervix uteri carcinoma. Archives of Cancer Biology and Therapy. 2021;2(1):8–14.

[2] Lu W, Lu C, Yu Z, Gao L. Chemoradiotherapy alone vs. chemoradiotherapy and hysterectomy for locally advanced cervical cancer: a systematic review and updated meta-analysis. Oncol Lett. 2021;21(2):1–1. 33552278 10.3892/ol.2020.12421 PMC7798101

[3] Li W, Liu P, Zhao W, Yin Z, Lin Z, Bin X, Lang J, Chen C. Effects of preoperative radiotherapy or chemoradiotherapy on postoperative pathological outcome of cervical cancer–from the large database of 46,313 cases of cervical cancer in China. Eur J Surg Oncol. 2020;46(1):148–54. 31623897 10.1016/j.ejso.2019.09.188

[4] Federico A, Anchora LP, Gallotta V, Fanfani F, Cosentino F, Turco LC, Bizzarri N, Legge F, Teodorico E, Macchia G, Valentini V. Clinical impact of pathologic residual tumor in locally advanced cervical cancer patients managed by chemoradiotherapy followed by radical surgery: a large, multicenter, retrospective study. Ann Surg Oncol. 2022;29(8):4806–14. 35355131 10.1245/s10434-022-11583-4 PMC9246767

[5] Ferrandina G, Legge F, Fagotti A, Fanfani F, Distefano M, Morganti A, Cellini N, Scambia G. Preoperative concomitant chemoradiotherapy in locally advanced cervical cancer: safety, outcome, and prognostic measures. Gynecol Oncol. 2007;107(1):S127–32. 17727936 10.1016/j.ygyno.2007.07.006

[6] Mancuso S, Smaniotto D, Panici PB, Favale B, Greggi S, Manfredi R, Margariti PA, Morganti AG, Scambia G, Tortoreto F, Valentini V. Phase I-II trial of preoperative chemoradiation in locally advanced cervical carcinoma. Gynaecol Oncol. 2000;78(3):324–8. 10.1006/gyno.2000.5862 10985888

[7] Mariagrazia D, Anna F, Gabriella F, Francesco F, Daniela S, Alessio M, Giovanni S. Preoperative chemoradiotherapy in locally advanced cervical cancer: long-term outcome and complications. Gynaecol Oncol. 2005;99(3):S166–70. 10.1016/j.ygyno.2005.07.074 16150482

[8] Fanfani F, Fagotti A, Ferrandina G, Raspagliesi F, Ditto A, Cerrotta AM, Morganti A, Smaniotto D, Scambia G. Neoadjuvant chemoradiation followed by radical hysterectomy in FIGO Stage IIIB cervical cancer: feasibility, complications, and clinical outcome. Int J Gynecol Cancer. 2009;19(6):1119–24. 19820379 10.1111/IGC.0b013e3181a8b08f

[9] Landoni F, Colombo A, Milani R, Placa F, Zanagnolo V, Mangioni C. Randomized study between radical surgery and radiotherapy for the treatment of stage IB–IIA cervical cancer: 20-year update. J Gynaecol Oncol. 2017;28(3):e34. 10.3802/jgo.2017.28.e34 PMC5391393 28382797

[10] Cetina L, Garcia-Arias A, Candelaria M, Cantú D, Rivera L, Coronel J, Bazan-Perkins B, Flores V, Gonzalez A, Dueñas-González A. Brachytherapy versus radical hysterectomy after external beam chemoradiation: a non-randomized matched comparison in IB2-IIB cervical cancer patients. World J Surg Oncol. 2009;7(1):1–8. 19220882 10.1186/1477-7819-7-19 PMC2649933

[11] Cetina L, González-Enciso A, Cantú D, Coronel J, Pérez-Montiel D, Hinojosa J, Serrano A, Rivera L, Poitevin A, Mota A, Trejo E. Brachytherapy versus radical hysterectomy after external beam chemoradiation with gemcitabine plus cisplatin: a randomized, phase III study in IB2–IIB cervical cancer patients. Ann Oncol. 2013;24(8):2043–7. 23609186 10.1093/annonc/mdt142

[12] Fanfani F, Vizza E, Landoni F, De Iaco P, Ferrandina G, Corrado G, Gallotta V, Gambacorta MA, Fagotti A, Monterossi G, Perrone AM. Radical hysterectomy after chemoradiation in FIGO stage III cervical cancer patients versus chemoradiation and brachytherapy: Complications and 3-years survival. Eur J Surg Oncol. 2016;42(10):1519–25. 27241922 10.1016/j.ejso.2016.05.011

[13] Hass P, Eggemann H, Costa SD, Ignatov A. Adjuvant hysterectomy after radiochemotherapy for locally advanced cervical cancer. Strahlenther Onkol. 2017;193(12):1048–55. 28660291 10.1007/s00066-017-1174-1

[14] Kol KV, Ebisch R, Piek J, Beugeling M, Vergeldt T, Bekkers R. Adjuvant Hysterectomy for Cervical Cancer patienys treated with chemoradiation therapy. A systematic review on the pathology-proven residual disease rate. Cancers (basel). 2021;13(24):6190. 34944810 10.3390/cancers13246190 PMC8699574

[15] Wang N, Li WW, Li JP, Liu JY, Zhou YC, Zhang Y, Hu J, Huang YH, Chen Y, Wei LC, Shi M. Comparison of concurrent chemoradiotherapy followed by radical surgery and high-dose-rate intracavitary brachytherapy: a retrospective study of 240 patients with FIGO stage IIB cervical carcinoma. OncoTargets Ther. 2014:91–100. 10.2147/OTT.S52710 PMC3888351 24421644

